# A Novel Polymer-Encapsulated Multi-Imaging Modality Fiducial Marker with Positive Signal Contrast for Image-Guided Radiation Therapy

**DOI:** 10.3390/cancers16030625

**Published:** 2024-01-31

**Authors:** Li Wang, Jeremiah Sanders, John F. Ward, Stephen R. Lee, Falk Poenisch, David Michael Swanson, Narayan Sahoo, Xiaorong Ronald Zhu, Jingfei Ma, Rajat J. Kudchadker, Seungtaek L. Choi, Quynh-Nhu Nguyen, Lauren L. Mayo, Shalin J. Shah, Steven J. Frank

**Affiliations:** 1 Department of Experimental Radiation Oncology, The University of Texas MD Anderson Cancer Center, Houston, TX 77030, USA; lwang6@mdanderson.org; 2 Department of Imaging Physics, The University of Texas MD Anderson Cancer Center, Houston, TX 77030, USA; sanders.jeremiah@mayo.edu (J.S.); jma@mdanderson.org (J.M.); 3 Department of Urology, The University of Texas MD Anderson Cancer Center, Houston, TX 77030, USA; jfward@mdanderson.org; 4 Department of Interventional Radiology, The University of Texas MD Anderson Cancer Center, Houston, TX 77030, USA; srlee@mdanderson.org; 5 Department of Radiation Physics, The University of Texas MD Anderson Cancer Center, Houston, TX 77030, USA; fpoenisch@mdanderson.org (F.P.); nsahoo@mdanderson.org (N.S.); xrzhu@mdanderson.org (X.R.Z.); rkudchad@mdanderson.org (R.J.K.); 6 Department of Biostatistics, The University of Texas MD Anderson Cancer Center, Houston, TX 77030, USA; dmswanson@mdanderson.org; 7 Department of Radiation Oncology, The University of Texas MD Anderson Cancer Center, Houston, TX 77030, USA; stchoi@mdanderson.org (S.L.C.); qnnguyen@mdanderson.org (Q.-N.N.); llmayo@mdanderson.org (L.L.M.); sjshah@mdanderson.org (S.J.S.)

**Keywords:** radiation therapy, prostate cancer, magnetic resonance imaging, fiducial marker, NOVA

## Abstract

**Simple Summary:**

External-beam radiotherapy (EBRT) is the standard of care for prostate cancer (PCa). Precision is critical to target the intraprostatic dominant lesion(s) and limit treatment-related toxicity from unnecessary irradiation of surrounding normal tissues. Currently, to achieve this goal, fiducial markers (FMs) are used in EBRT planning and treatment delivery. However, some of the features of current FMs (e.g., poor visibility on MRI, substantial CT and MR artifacts, radiation dose perturbations) affect treatment accuracy. The aim of our study was to evaluate the performance of a novel FM (NOVA) in phantoms and in a patient. We found that NOVA FMs were positively visualized on ultrasound (US), kV X-ray, CT, and MR images, had reduced CT and MR artifacts, and caused less proton dose perturbations than Gold Anchor and BiomarC (carbon) FMs. Our findings indicate the value of NOVA FMs in daily treatment positioning, MRI/CT co-image registration, and MRI-based treatment-plan generation for patients with PCa undergoing EBRT.

**Abstract:**

Background: Current fiducial markers (FMs) in external-beam radiotherapy (EBRT) for prostate cancer (PCa) cannot be positively visualized on magnetic resonance imaging (MRI) and create dose perturbation and significant imaging artifacts on computed tomography (CT) and MRI. We report our initial experience with clinical imaging of a novel multimodality FM, NOVA. Methods: We tested Gold Anchor [G-FM], BiomarC [carbon, C-FM], and NOVA FMs in phantoms imaged with kilovoltage (kV) X-rays, transrectal ultrasound (TRUS), CT, and MRI. Artifacts of the FMs on CT were quantified by the relative streak artifacts level (rSAL) metric. Proton dose perturbations (PDPs) were measured with Gafchromic EBT3 film, with FMs oriented either perpendicular to or parallel with the beam axis. We also tested the performance of NOVA-FMs in a patient. Results: NOVA-FMs were positively visualized on all 4 imaging modalities tested. The rSAL on CT was 0.750 ± 0.335 for 2-mm reconstructed slices. In F-tests, PDP was associated with marker type and depth of measurement (*p* < 10^−6^); at 5-mm depth, PDP was significantly greater for the G-FM (12.9%, *p* = 10^−6^) and C-FM (6.0%, *p* = 0.011) than NOVA (4.5%). EBRT planning with MRI/CT image co-registration and daily alignments using NOVA-FMs in a patient was feasible and reproducible. Conclusions: NOVA-FMs were positively visible and produced less PDP than G-FMs or C-FMs. NOVA-FMs facilitated MRI/CT fusion and identification of regions of interest.

## 1. Introduction

External-beam radiation therapy (EBRT) is a standard-of-care approach for the management of prostate cancer, and magnetic resonance imaging (MRI) is increasingly used at every step of the treatment process, from diagnosis and biopsy to simulation, treatment planning, and treatment delivery. Precise imaging is crucial for dose escalation to the dominant tumor and for ultra-hypofractionated RT. Treatment-related toxicity from unnecessary irradiation of surrounding normal tissues continues to be a major concern. To mitigate radiation-related toxicity while improving tumor control, several imaging modalities are being used during EBRT for prostate cancer [1]. MRI is being used during diagnosis for fusion scan-directed biopsy of the dominant lesion, and during simulation for radiation treatment planning. Ultrasound is used for biopsy, fiducial marker (FM) placement, and placement of hydrogel spacers. Computed tomography (CT) is often used for radiation treatment planning and daily alignment, whereas kV X-ray imaging is commonly used for daily alignment and treatment verification. Use of MRI/CT fusion can improve the resolution of the pelvic anatomy but is limited by problems with image co-registration and the visibility of, or artifacts from, implanted FMs. Current FMs cannot be seen with positive contrast on MRI, and metallic FMs create CT artifacts, MR susceptibility artifacts, and radiation dose perturbations.

The accuracy and reproducibility of daily patient positioning can be assessed by kV imaging, CT, or MRI. Daily setup positioning images are captured before delivery of each radiation fraction and compared with treatment planning images according to bony or soft tissue anatomic markers; patient alignment and positioning are adjusted based on matches with these images. However, the accuracy of daily patient alignment is reduced by inter- and intra-observer variability in identifying and assigning anatomic markers [2] and by changes in the locations of those markers resulting from organ motion or filling status of the bladder and rectum, which can affect patient positioning [3] and increase the risks of normal tissue damage and suboptimal tumor control. Fiducial markers (FMs) have been used to facilitate daily image-guided positioning for RT, especially intraprostatic FMs for men with prostate cancer [4]. Daily treatment positioning is aligned by matching the FMs on both pretreatment (setup) images and the treatment-planning digitally reconstructed radiograph images. Use of FMs can aid in overcoming the poor image contrast between the prostate and surrounding normal tissues on kV or CT images [1,5,6,7,8,9]. Moreover, because FM-based treatment positioning is faster and more accurate than methods without FMs [10], FMs can be useful for ensuring precise RT for prostate cancer.

The precision and accuracy of treatment planning can also be enhanced by the use of MRI, which provides better soft tissue contrast than CT. The advantages of MRI-guided over CT-guided stereotactic body RT in reducing treatment-related acute toxicities for patients with prostate cancer were recently demonstrated in a phase 3 randomized clinical trial [11]. In that trial, patients treated with MRI-guided (versus CT-guided) stereotactic body RT experienced significantly fewer urinary and bowel grade ≥ 2 toxicities including urinary frequency, urinary retention, diarrhea, and proctitis [11]. Because most current radiation treatment planning systems are CT-based, the advantages of MRI for EBRT can be achieved only through MRI/CT image co-registration. Use of fused MRI/CT images can improve the accuracy of radiation dose distribution over CT alone, both for treatment of prostate tumors and for protection of normal tissues. Notably, the use of FMs can be also helpful in MRI/CT-based image registration for organ delineation [12,13] and for generating precise target volume margins [14,15].

Several types of FMs are in current use in clinical applications. Differences in the materials used and their size translate to differences in their visibility and the artifacts they produce on medical images [16,17,18,19,20]. Some FMs can cause large (10–38%) photon or proton radiation dose perturbations [21,22,23,24,25,26], which can lead to inadequate doses downstream of the FMs. Metallic (e.g., gold) FMs are typically used for photon therapy, and non-metallic FMs (e.g., carbon) for proton therapy, for prostate cancer. Although carbon FMs produce fewer proton dose perturbations than gold FMs, both can cause streak artifacts on kV and CT images [27] and signal void artifacts on MR images [17]. These artifacts can influence the accuracy of target delineation in MRI and CT as well as affecting the CT values around the FMs, with substantial effects on treatment plan accuracy [21,28], daily patient positioning, and consequently tumor control and normal-tissue sparing [27]. Al-though the use of MRI/CT image co-registration can improve the precision of radiation treatment plans, neither gold nor carbon FMs are positively visible on MRI; gold FMs have large MR susceptibility artifacts; and carbon FMs cannot be accurately localized on MR images, all of which limit the reproducibility of patient positioning and have thus far precluded the use of MRI/CT image fusion in community settings.

The ideal FM for reproducible, accurate daily positioning and treatment planning for patients with prostate cancer would be positively visible across several imaging modalities, such as transrectal ultrasound (TRUS), kV X-ray, CT, and MRI, have minimal image artifacts, and cause minimal radiation dose perturbations [27]. NOVA (C4 Imaging, LLC, Bellaire, TX) is an FDA-approved non-metallic FM that has all the ideal FM attributes such as positive signal visibility on both MRI and CT with minimal artifacts and reduction in radiation dose perturbation. Use of NOVA FMs can contribute to more accurate radiation treatment planning and image-guided delivery. The NOVA FM consists of a sealed polyether ether ketone (PEEK) polymer capsule containing a solution of cobalt chloride and N-acetyl cysteine. A fixation device along with zirconium oxide is also sealed within the FM. In the current study, we investigated NOVA for its visibility on US, kV X-ray, CT, and MR images; for imaging artifacts on CT images; and for proton dose perturbations. We also describe our early experience with using NOVA FMs for daily treatment positioning, MRI/CT co-image registration, and treatment-plan generation for a patient undergoing EBRT for prostate cancer.

## 2. Materials and Methods

### 2.1. Fiducial Markers

**Figure A1:**
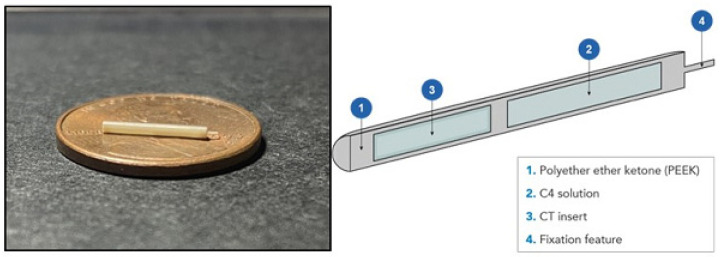
A schematic diagram illustrating the configuration and composition of the NOVA fiducial marker (10 mm length, 1 mm outer diameter).

Three FMs were tested for visibility on imaging, artifacts on CT, and proton dose perturbations: the novel NOVA FM (Figure A1, 1.0 × 9.8 mm; C4 imaging, Bellaire, TX, USA), the Gold Anchor FM (0.38 × 8.4 mm; Naslund Medical AB, Chicago, IL, USA), and the BiomarC FM (Carbon FM, 1 × 3 mm; Carbon Medical Technologies, Inc., St Paul, MN, USA).

### 2.2. Phantoms and Fiducial Marker Implantation

Tissue-equivalent US prostate phantoms (Model 404A, Computerized Imaging Reference Systems [CIRS], Inc., Norfolk, VA, USA) were used for FM imaging and artifact measurements. The FMs were implanted into the phantoms with 18 G needles and imaged as described below.

### 2.3. Imaging Acquisition for Phantom

TRUS images were acquired in 5-mm slices consistent with the treatment planning process for brachytherapy by using a BCL 10-5 curvilinear transducer (Sonoscape Medical Corp, Shenzhen, China). For all other types of imaging, the phantom with FMs implanted was positioned at the isocenter of the scanner. The kV images were obtained with a proton therapy system (Hitachi, Ltd., Chiyoda-ku, Tokyo, Japan) and a TrueBeam On-Board Imager (Varian Medical Systems, Palo Alto, CA, USA). The following institutional protocols for pelvic imaging acquisition were used: 60 kVp, 500 mA tube current, and 31 mAs for anterior–posterior (AP) images; and 100 kVp, 400 mA tube current, and 157 mAs for lateral images. CT images were obtained with a 16-slice CT simulation scanner (Siemens Healthineers, Erlangen, Germany) according to a clinical protocol for pelvic scans (120 kVp, 330 mA tube current, 0.5 s revolution time, 0.5625 spiral pitch factor, 2.5-mm reconstruction slice thickness, and 0.9766 mm pixel spacing) [18,27]. MR images were obtained with a clinical 1.5 T scanner (Siemens Healthineers, Erlangen, Germany) with two 18-channel external coil arrays. MR images of the phantom were acquired with a fully balanced steady state free precession (bSSFP) pulse sequence (Siemens CISS).

### 2.4. Artifact Measurement on CT

FMs used in clinical practice for photon and proton radiation (e.g., Gold Anchor, BiomarC) can generate artifacts after CT image reconstruction that appear as streaks or signal voids in areas adjacent to the implanted FMs, which can reduce the accuracy of radiation treatment plans [21,28]. Artifacts from the NOVA FMs on CT images were measured and quantified in terms of the relative streak artifacts level metric (rSAL) and the total variation function (*TV*[*x*]) [17,18], as follows. Briefly, the phantom was first imaged without the FMs (to derive the *X_ref_* term in Equation (1)), after which the FMs were implanted and the phantom imaged with the FMs in place (to derive the Xartifact term). A 30 mm × 30 mm region of interest was chosen, with an FM at the center, and the metrics derived via Equations (1) and (2):

(1) $$rSAL=\frac{\left|TV\left(X_{artifact}\right)-TV\left(X_{ref}\right)\right|}{TV\left(X_{ref}\right)}$$

(2) $$TV\left(x\right)=\sum_{i,j}\sqrt{{\left(x_{i+1,j}-x_{i,j}\right)}^{2}+{\left(x_{i,j+1}-x_{i,j}\right)}^{2}}$$

Larger *TV*[*x*] values represent higher levels of streak artifacts. Artifact levels were further quantified in terms of the relative standard deviation (rSD) between the image with the FM (Sartifact; the SD of the Xartifact image values) and the image without the FM (Sref; the SD of the *X_ref_* image values), as shown in Equation (3). rSD values close to 1.0 represent minimal artifacts, whereas larger rSD values represent larger artifacts.

(3) $$\sigma_{ref}^{artifact}=\frac{\sigma_{artifact}}{\sigma_{ref}}$$

### 2.5. Proton Radiation Dose Perturbation Assessment

**Figure A2:**
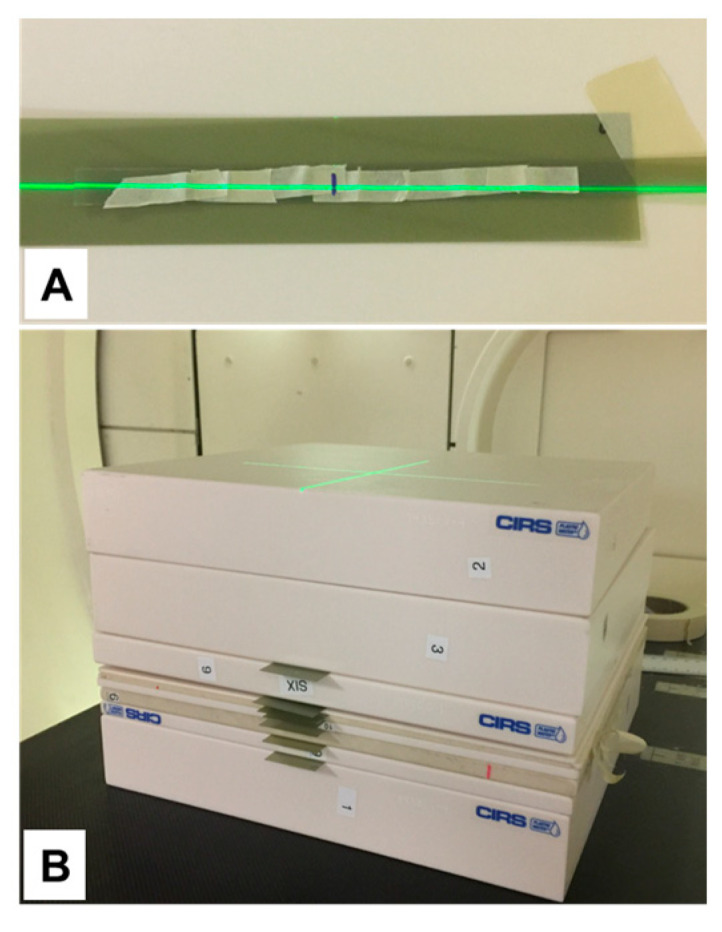
Fiducial marker arrangement for proton dose perturbation study. Fiducial markers were arranged inside plastic water-equivalent material at the center of a passively scattered, spread-out Bragg peak (160 MeV, range 16 cm, SOBP 10 cm). The beam is coming from the top. The first film is for control, and the next EBT3 films are located at 0, 2, 5, 10, 20, 30, and 40 mm downstream of the markers (panel (**B**)). (Panel (**A**)) shows the preparation of the fiducial onto the EBT3 film: the fiducials are attached with masking tape onto a strip of about 1 cm, which is then overlayed flat onto the 3 × 20 cm wide EBT3 film strip (shown perpendicular arrangement).

The proton radiation dose perturbations caused by the NOVA, Gold Anchor, and BiomarC FMs were evaluated with a proton therapy system (Hitachi, Ltd., Chiyoda-ku, Tokyo, Japan) [18,27]. In clinical practice, tumors are normally positioned inside the spread-out Bragg peak (SOBP) during proton therapy; for this study, the FMs were placed inside plastic water-equivalent material (CIRS, Norfolk, VA) at the center of a passively scattered SOBP (160 MeV, range 16 cm, SOBP 10 cm) (Figure A2). The dose shadows created by the FMs were measured with radiochromic film (Gafchromic EBT3, International Specialty Products, Wayne, NJ, USA) placed within the SOBP at 0, 2, 5, 10, 20, 30, and 40 mm downstream from the FMs. The FMs were oriented perpendicular to or parallel with the beam axis. The films were scanned with an Epson 11000XL (Epson, Long Beach, CA, USA) flatbed scanner at 300 dpi and analyzed further with ImageJ software (NIH, version 1.52a). All films were scanned in 48-bit color (RGB) 72 h after irradiation and analyzed with the red channel. To generate the optical-density-to-dose curve, calibration measurements were obtained at a water-equivalent depth of 27.5 cm with proton radiation doses ranging from 1 Gy to 6 Gy on a single sheet of film (cut into several pieces). The background optical density value was obtained from a non-irradiated piece of film.

### 2.6. Artemis Computer-Assisted Fiducial Marker Implantation for Intraprostatic Tumor Localization

We also analyzed the performance of NOVA and Gold Anchor FMs in a patient with locally recurrent prostate cancer. The NOVA (1.0 × 9.8 mm) and Gold Anchor (0.38 × 8.4 mm) FMs were implanted by using an Artemis (Eigen, Green Valley, CA, USA) computer-assisted robotic needle guidance system to outline the intraprostatic tumor, as described elsewhere [29,30].

### 2.7. Acquiring Patient Images

The kV portal images were acquired with a Hitachi proton therapy system and Varian TrueBeam; CT images were acquired with a Siemens simulation scanner. MR images were obtained with a fully-balanced bSSFP pulse sequence on a 3.0 T scanner (Siemens CISS). MRI/CT co-image fusion registration images based on the NOVA FMs were used for EBRT planning. MRI was used to contour the prostate, seminal vesicles, rectum, bladder, and external urethral sphincter. Daily imaging was used to verify and re-align the patient’s position for EBRT.

### 2.8. MRI/CT Image Fusion

Fusion of the MRI and CT images was done as described elsewhere [31]. Briefly, both MR and CT images were acquired within an hour to minimize differences caused by bladder and rectal filling. The RayStation treatment planning system was used for image fusion, contouring, and dosimetry calculations. Bony and soft tissue landmarks (specifically the prostate) were used for manual fusion of the axial CT and axial bSSFP MR images. Signals from the NOVA FMs on the CT and bSSFP MR images were used to improve the fusion. Thereafter, the prostate, seminal vesicles, bladder, urethra, and rectum were contoured on the CT/MRI fused image.

### 2.9. Statistical Analysis

We fit regression models of the dose perturbation measurements (in percentages) on distance and within strata—that is, whether the marker was perpendicular to or parallel with the beam. Non-linearity in the distance–perturbation relationship was accounted for by including a squared term of distance. Models were compared by using F-tests, and the relative magnitude of perturbations at specific distances was described by parameter estimate contrasts.

## 3. Results

### 3.1. NOVA Marker Visibility in Phantom

**Figure A3:**
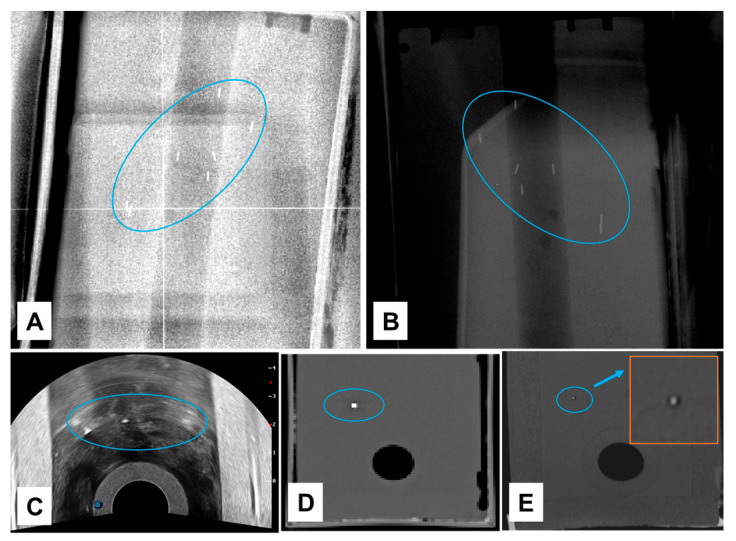
Visibility of NOVA markers (blue circles) implanted in a tissue-equivalent phantom. (**A**,**B**) kV images from a Hitachi proton therapy system (**A**) or a Varian TrueBeam (**B**); (**C**) ultrasonographic image; (**D**) CT image (window width 1500, window level 250); or (**E**) magnetic resonance image. The scan showed in (**E**) was acquired by a clinical 1.5 T scanner with a fully bSSFP pulse sequence. The orange box in panel E represents enlarged view of the blue circle in panel E. NOVA fiducial markers were positively visualized in all imaging modalities tested.

Images of an in vitro phantom acquired with clinical scanners confirmed that the same NOVA FMs were positively visualized without difficulty by all four imaging modalities used for image-guided RT in prostate cancer: kV (Figure A3A,B), TRUS (Figure A3C), CT (window width 1500, window level 250; Figure A3D), and MRI (Figure A3E).

### 3.2. NOVA Marker Artifacts on CT Images

CT images of five NOVA FMs implanted in the tissue-equivalent phantom were analyzed and quantified for marker artifacts by rSAL and rSD. The rSAL of the NOVA markers was 0.682 ± 0.305 (range 0.506–0.828) for 1-mm reconstructed slices and 0.750 ± 0.335 (range 0.575–0.868) for 2-mm reconstructed slices. The corresponding rSD values were 2.139 ± 0.957 (range 1.814–2.563) for the 1-mm slices and 1.949 ± 0.871 (range 1.760–2.459) for the 2-mm slices.

### 3.3. Gold Anchor, BiomarC, and NOVA Proton Radiation Dose Perturbations

**Figure 1:**
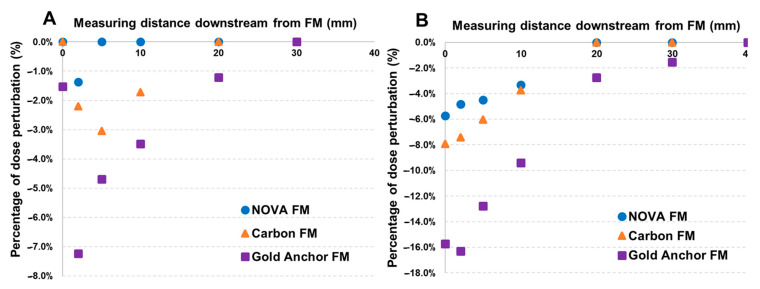
Proton radiation dose perturbation by 3 types of fiducial markers (FMs). Gold Anchor, BiomarC, and NOVA FMs were arranged (**A**) perpendicular to or (**B**) parallel with the proton beam axis. Proton dose perturbation by the different FMs was measured at various depths downstream of the markers. The maximum dose perturbations of Gold Anchor, BiomarC, and NOVA FMs placed perpendicular to the beam were 7.2% (30 mm depth), 3.0% (20 mm depth), and 1.4% (5 mm depth) respectively; corresponding values for FMs placed in parallel were 16.3% at 40 mm, 7.9% 20 mm, and 5.7% at 20 mm.

The proton dose perturbations from Gold Anchor, BiomarC carbon, and NOVA FMs at various depths downstream of the markers, with the markers in different orientations, are shown in Figure 1. Proton dose perturbation was associated with marker type, orientation, and the depth of measurement. For all FMs, proton dose perturbation increased with the distance (depth) from the FMs before gradually decreasing to zero. Dose perturbation was lower when the marker was perpendicular to the beam (Figure 1A) relative to parallel with the beam (Figure 1B). Gold Anchor FMs produced the greatest maximum dose perturbation (7.2% perpendicular, 16.3% parallel) and the deepest dose perturbation depth (30 mm perpendicular, 40 mm parallel) relative to the BiomarC and NOVA FMs. Compared with the BiomarC (carbon) FMs, the NOVA markers produced lower maximum dose perturbations (1.4% vs. 3.0% perpendicular, 5.7% vs. 7.9% parallel) and shallower perturbation depths (5 vs. 20 mm perpendicular).

An F-test of perturbation by distance and distance-squared, with separate intercepts for anchor type, was rejected in favor of models with additional, separate linear slopes for each anchor type for the parallel marker stratum (*p* < 10^−6^). This result is interpreted as meaning that each marker type has distinct characteristics with respect to perturbation by distance. A comparison of NOVA vs. Gold and NOVA vs. Carbon at a distance of 5 mm, where perturbations tended to be greater, yielded significant differences in the expected amount of perturbation of 8.6% (*p* = 10^−6^) (vs. Gold) and 1.6% (*p* = 0.011) (vs. Carbon), and NOVA had the smallest expected perturbation.

For the perpendicular stratum, an F-test of models with distance and distance-squared terms was rejected in favor of one with additional separate intercepts by anchor type (*p* = 0.026). Because of lower-than-expected perturbations for anchors at distances of <3 mm among this stratum, we explored a separate intercept term for these observations. This term was not included because of a lack of fit improvement in the model with separate distance-anchor intercepts and distance-squared term (*p* = 0.92). Similarly, no evidence was found for separate slopes in addition to intercepts among the perpendicular data (*p* = 0.28). The difference between the NOVA and Gold Anchor intercepts in this model was 0.028, suggesting that NOVA had approximately 2.8% less perturbation than Gold. The difference between NOVA and Carbon was smaller at 0.9%, with NOVA having modestly less perturbation.

### 3.4. Visibility of NOVA Markers in a Clinical Case

**Figure 2:**
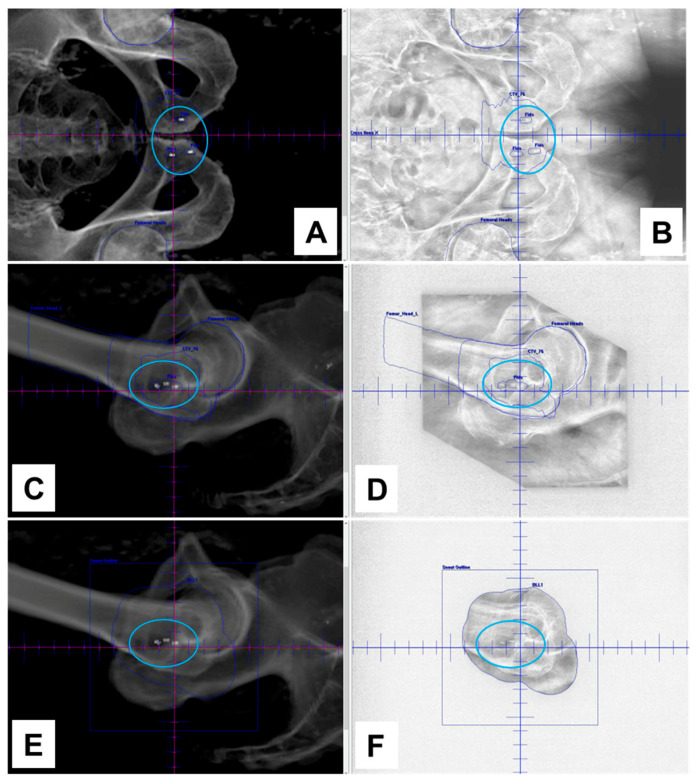
Visibility of NOVA fiducial markers on kV images of a patient undergoing proton therapy for prostate cancer. Images were captured with a clinical proton therapy system. Panels (**A**,**C**,**E**) show digitally reconstructed images; panels (**B**,**D**) show image setup verification; panel (**F**) shows treatment positioning verification. NOVA fiducial markers were clearly visualized in both anteroposterior (panels (**A**,**B**)) and lateral (panels (**C**–**E**)) views.

**Figure 3:**
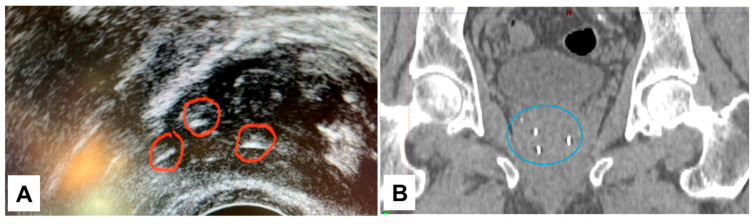
Visibility of NOVA fiducial markers on ultrasound (**A**), inside the red circles) and CT images ((**B**), inside the blue circle) of a patient receiving proton therapy for prostate cancer. The patient was positioned for CT simulation scanning. NOVA markers were clearly visible on the CT image (CT value = 2951 Hounsfield units).

**Figure 4:**
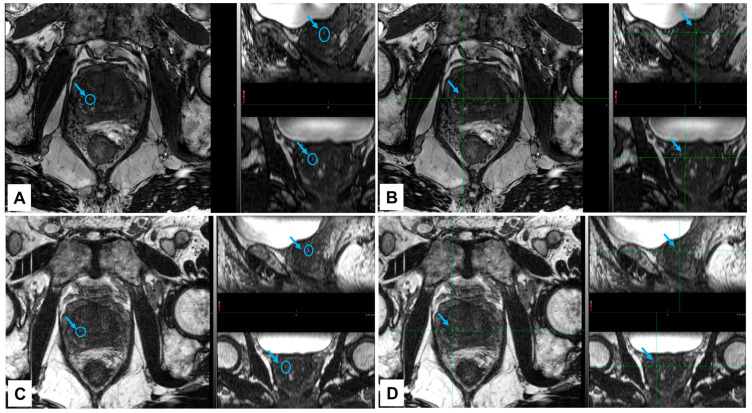
Visibility of NOVA fiducial markers on MR images of a patient receiving proton therapy for prostate cancer. The patient was positioned for MRI, and scans were obtained on a clinical 3.0 T scanner (panels (**A**,**B**)) and a 1.5 T scanner (panels (**C**,**D**)). MRI scans were acquired with a fully balanced steady state free precession (bSSFP) pulse sequence. NOVA markers were visualized as positive signal on both the 3.0 T images (panels (**A**,**B**); blue circles and blue arrows) and the 1.5 T images (panels (**C**,**D**); blue circles and blue arrows). Included in each panel are an axial image (acquired with a 15 cm field-of-view and 1.2 mm slice thickness) and two reformatted images (in the sagittal and coronal planes).

**Figure 5:**
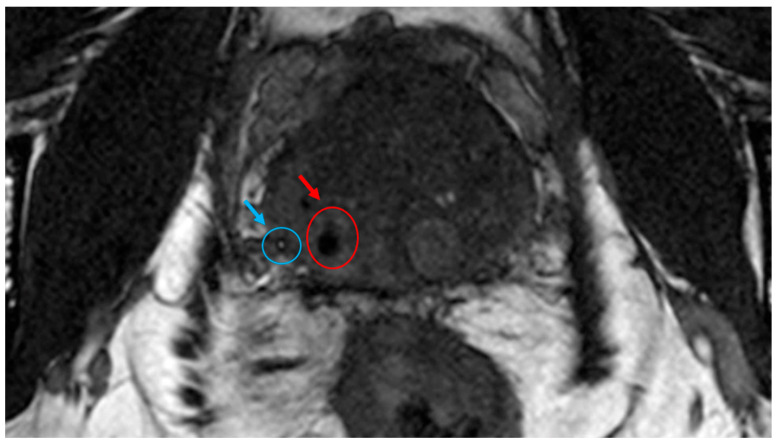
Visibility of NOVA and Gold Anchor fiducial markers on an MR image of a patient undergoing proton therapy for prostate cancer. NOVA markers (blue circle and blue arrow) are visualized as a positive signal, whereas Gold Anchor markers (red circle and red arrow) presented as a susceptibility artifact.

NOVA FMs were implanted around the tumor of a patient undergoing EBRT for localized prostate cancer. The NOVA markers were clearly visualized on kV images in both the AP (Figure 2A) and lateral (Figure 2C,E) digitally reconstructed treatment positioning setup images (Figure 2B,D), and in the verification-of-positioning images (Figure 2F). The NOVA markers were also positively visualized in the prostate on CT (Figure 3; CT value = 2951 HU) images, and on the MR images acquired with either the 3.0 T (Figure 4A,B) or 1.5 T (Figure 4C,D) MRI scanners, and showed smaller artifacts (Figure 5, blue circle) than the Gold Anchor FM (Figure 5, red circle).

**Figure 6:**
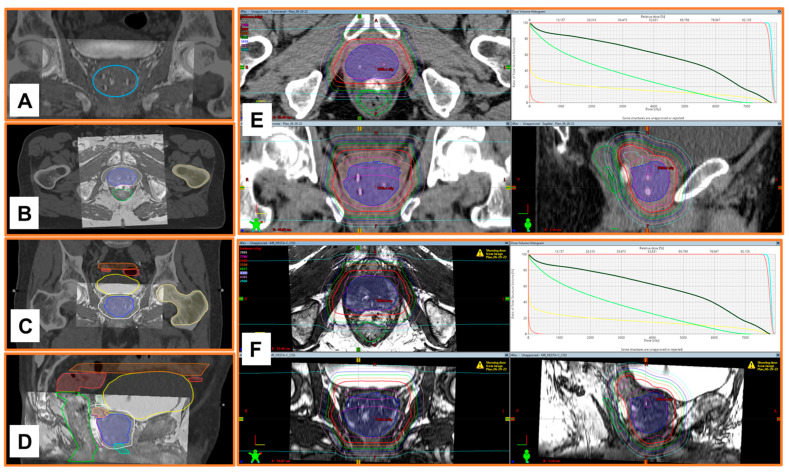
Use of NOVA markers for MRI/CT image fusion, contouring organs of interest, and generating radiation treatment plans. Pelvic MRI and CT images of a patient with prostate cancer were imported to the RayStation treatment planning system. Bony and soft tissue landmarks (specifically the prostate) were used to manually fuse axial CT and axial fully balanced steady state free precession (bSSFP) pulse sequence MR images. The positive NOVA fiducial marker signals from the CT and MR images were used to improve the fusion (panel ((**A**), inside the blue circle). The prostate, seminal vesicles, bladder, urethra, and rectum were contoured on the CT-MRI fused images (axial, panel (**B**); coronal, panel (**C**); sagittal, panel (**D**)). Panels (**E**,**F**) show proton radiation treatment plans generated based on CT only (**E**) and on MRI/CT image fusion (**F**).

Finally, we tested the feasibility of using NOVA FMs to facilitate MRI/CT image fusion in the same patient. The MR and CT images were fused and registered based on the location of the NOVA markers by the RayStation treatment planning system (Figure 6A). Both the target to be treated and the surrounding organs of interest (required to generate a radiation treatment plan) were distinctly contoured on the fused MRI/CT image (Figure 6B–D). Notably, the radiation treatment plan generated based on the MRI/CT image fusion (Figure 6F) provided more precise soft tissue anatomy than the one based on CT alone (Figure 6E).

## 4. Discussion

The aims of the study reported here were to (1) evaluate the visibility of the novel, FDA-approved FM NOVA on TRUS, kV, CT, and MR images; (2) evaluate artifacts of the NOVA markers on CT images; (3) determine proton dose perturbations from the NOVA, Gold Anchor, and BiomarC markers; and (4) describe our early experience with NOVA FMs for daily patient positioning, MRI/CT image fusion, and treatment planning for a patient receiving EBRT for prostate cancer. We found that the NOVA FMs were visible (as positive signal) on all images tested, with smaller artifacts than Gold Anchor FMs [17] and smaller proton dose perturbations than the gold and carbon markers. The NOVA FMs functioned effectively in daily patient positioning for EBRT, and the treatment plan generated for MRI/CT-fused images from NOVA FMs generated more clearly visualized anatomic structures than treatment plans generated from CT alone.

MR- versus CT-based contouring was shown to facilitate the generation of more precise and accurate treatment plans, and thus reduce radiation-related toxicities, in a phase 3 randomized clinical trial in men with prostate cancer [11]. This finding emphasizes the importance of MRI/CT image co-registration in generation of EBRT treatment plans, the precision of which relies on the use of effective FMs [12,13,14,15]. The positive visibility and limited artifacts for FMs in both applications (daily verification of treatment positioning and treatment planning) are important for ensuring the precision of RT, which in turn contributes to effective tumor control and less normal-tissue toxicity. Even though gold FMs for photon radiation, and carbon markers for proton radiation, have been widely used in clinical applications and appear as positive signals on kV and CT images, streak artifacts from these FMs on CT, and void artifacts on MRI, can introduce uncertainties for target and organ delineation. These uncertainties have been a longstanding concern for physicians, radiation physicists, and dosimetrists because they can negatively affect the accuracy of the dosimetry for radiation treatment planning, which in turn limits the application of MRI in community radiation oncology services. We determined here that NOVA FMs had positive contrast visibility in several imaging modalities, including MRI (Figure A3). Moreover, quantification of artifacts from the NOVA FMs on CT images [17] showed that the average rSALs were 0.682 ± 0.305 on 1-mm reconstructed slices and 0.750 ± 0.335 on 2-mm reconstructed slices; the corresponding rSD values were 2.139 ± 0.957 and 1.949 ± 0.872, respectively. These artifact levels are smaller than those reported for Gold Anchor FMs (rSAL of 0.57 and rSD of 3.9) [17]. Because artifacts may obscure the margins of the organs at risk, or obscure the primary tumor for tumor localization, our finding suggests that using NOVA markers may improve the accuracy of organ delineation and dose calculation, and thereby facilitate more precise treatment plans, over using gold FMs.

One concern regarding the use of FMs, especially gold and carbon, in RT for prostate cancer is their capacity for dose perturbation, with corresponding effects on the precision of radiation dose [18,21,22,24,27,32,33]. Indeed, the extent of dose perturbation caused by FMs can be up to 38% for protons [18,24,27,32]. In the current study, we used radiochromic film to quantify the proton dose perturbation of the three FMs (Figure 1). When the FMs were arranged perpendicular to the proton beam axis (as is done in clinical practice for prostate irradiation), the maximum dose perturbation was 7.2% for Gold Anchor (depth 30 mm), 3.0% for BiomarC (depth 20 mm), and 1.4% for NOVA FMs (depth 5 mm). The lower dose perturbation from NOVA FMs may translate to a more precise proton dose relative to the gold and carbon FMs. Notably, when the FMs were arranged parallel with the proton beam (to simulate rotation of the implanted FMs after implantation or during treatment in clinical practice), the maximum dose perturbations were considerably higher at 16.3% for Gold Anchor (depth 40 mm), 7.9% for BiomarC (depth 20 mm), and 5.7% for NOVA (depth 10 mm). Because the clinical tolerance for dose inhomogeneity in previous studies is 10% [24,32], these findings suggest that using NOVA FMs can maintain proton dose accuracy even under FM rotation compared with the two other FMs tested [18,27].

The insights gained from our in vitro phantom studies of marker visibility, CT artifacts, and dose perturbation offer valuable information despite their inherent limitations owing to the interdependence of these factors [18]; moreover, any small air gaps in vitro can cause errors in parameter measurements, and patient anatomy is more heterogeneous than a tissue-equivalent phantom. To overcome these limitations, we also tested NOVA FMs in a patient with prostate cancer to evaluate the clinical application in EBRT for prostate cancer. The NOVA markers were clearly visible on kV images captured by a clinical proton therapy system at all stages of patient alignment and treatment planning, including simulation digital reconstruction of the images and verification of imaging setup and treatment positioning (Figure 2), suggesting that NOVA FMs can enable daily reproducibility of treatment position between fractions. The NOVA FMs were also clearly visible on other imaging modalities used for radiation treatment planning, including CT and MRI, suggesting that NOVA FMs are effective for reducing errors in organ delineation and ensuring the accuracy of treatment planning (Figure 3). Notably, unlike other FMs in common use in prostate clinics (gold or carbon) [16], NOVA FMs appeared as positive contrast and were clearly visible on both 3.0 T and 1.5 T MR images captured with typical clinical sequences (bSSFP) (Figure 4) without the large susceptibility artifacts on MR images characteristic of the gold markers (Figure 5) [16,34,35]. This important feature allows more precise, fiducial-based MRI/CT image fusion compared with use of bony and soft tissue landmarks, with correspondingly more accurate dosimetric treatment plans (Figure 6). Our early experience with using NOVA FMs to treat a patient with prostate cancer suggests that NOVA FMs may provide superior precision and warrant further study in additional patients.

To the best of our knowledge, we are the first to report positive contrast visibility in four imaging modalities used in image-guided RT; smaller artifacts on CT; smaller radiation dose perturbations; and use of NOVA FMs for treatment planning and daily positioning alignment in a patient with prostate cancer. Nevertheless, our study had several limitations. First, the long-term stability of markers and their position within the prostate are crucial for precise positioning and dose accuracy, especially for fractionated RT [27,36,37,38,39,40,41,42]. We have yet to study intraprostate migration and rotation of NOVA FMs. However, the NOVA FM has a fixation device at the tip of the marker to minimize the risks of rotation or migration, and future studies with additional patients are needed to further evaluate these issues. Second, unlike previous systematic comparisons of FMs of various materials, shapes, and sizes [16,17,18], we tested only two FMs, one gold- and the other carbon-based, both in clinical use at our institution, and compared them in terms of artifacts and proton dose perturbations against the NOVA FMs. Our initial focus has been on the potential clinical advantages of using NOVA FMs to facilitate MRI/CT-based precision RT for patients with prostate cancer. A systematic comparison of all features in other commercially available FMs will be the subject of a future report.

## 5. Conclusions

In summary, we evaluated several aspects of a novel, commercially available, FDA-approved multimodality fiducial marker, NOVA, for its visibility on four different imaging modalities commonly used for RT planning in prostate cancer: kV, US, CT, and MR. The NOVA markers were clearly and positively visible on all four modalities, including MRI. Our quantification of marker-induced artifacts showed that the NOVA FMs produced smaller artifacts than the Gold Anchor FMs, and the NOVA FMs were also found to produce smaller proton radiation dose perturbations than the Gold Anchor and BiomarC FMs. NOVA is the first non-metallic, MRI-positive signal fiducial marker that significantly reduces dose perturbation while minimizing artifacts in CT and MRI imaging processes, thereby contributing to more accurate radiation therapy treatment planning. These findings demonstrate the potential advantages of NOVA FMs over metallic-based markers for improved RT in clinical practice.

## Data Availability

The raw data supporting the conclusions of this article will be made available by the authors on request.
